# Targeted removal of the 16S rRNA anti-Shine–Dalgarno sequence by a *Mycobacterium tuberculosis* MazF toxin

**DOI:** 10.1016/j.jbc.2025.110323

**Published:** 2025-05-30

**Authors:** Timothy W. Sherrier, Valdir C. Barth, Jason M. Schifano, Julia R. Greendyk, Nancy A. Woychik

**Affiliations:** Department of Biochemistry and Molecular Biology, Rutgers University, Robert Wood Johnson Medical School, Piscataway, New Jersey, USA

**Keywords:** toxin–antitoxin systems, tuberculosis, ribosome, RNA, endoribonuclease

## Abstract

The genome of the bacterial pathogen that causes tuberculosis, *Mycobacterium tuberculosis* (Mtb), encodes an inexplicably high number of type II toxin–antitoxin (TA) systems. Because this ancient pathogen has evolved to resist clearance by antibiotics and the host immune system, its TA systems are thought to participate in the survival of these stresses. Of the ∼70 Mtb type II TA systems, 10 MazEF family members have been previously identified, yet the precise cellular target of only one of these MazF toxins is known. Here, we demonstrate that the Rv3098A gene encodes an 11th MazF paralog in Mtb (MazF-mt11, MazF11). As with all MazF toxins, MazF-mt11 acts as a single-strand, sequence-specific endoribonuclease. We first performed primer extension on the large single-stranded MS2 enterobacteriophage RNA substrate after incubation with recombinant MazF-mt11 to identify a single toxin cleavage site between C↓A. We then further pinpointed the boundaries of the MazF-mt11 cleavage consensus sequence as C↓ACCU using *Escherichia coli* MORE (Mapping by Overexpression of an RNase in *E. coli*) RNA-seq. Finally, we enlisted 5′-OH RNA-seq to reveal 16S rRNA in the 30S ribosomal subunit as the only MazF-mt11 RNA target in mycobacteria. In fact, the single cleavage site in C↓ACCU maps just before the anti-Shine–Dalgarno sequence at the 3′ end of 16S rRNA. Targeted removal of the anti-Shine–Dalgarno sequence by MazF-mt11 leads to nearly complete inhibition of protein synthesis, consistent with its important role in directing ribosomes to translation start codons in leadered mRNAs. The accompanying growth arrest phenotype suggests that MazF-mt11 may participate in establishment of the nonreplicating persistent state in Mtb.

It is estimated that over one-quarter of the world’s population is infected with *Mycobacterium tuberculosis* (Mtb), the bacterial pathogen that causes tuberculosis (TB) (1, 2). Even though 95% of those infected are asymptomatic and noninfectious (*i.e.*, have a latent TB infection), 5% are predicted to progress to active, contagious TB within 2 years (2, 3). Following worldwide disruption from the COVID-19 pandemic, TB once again causes the most deaths (1.25 million) per year by a single infectious agent (1). This ancient pathogen has adapted to evade clearing by the host immune system by reprogramming its physiology in response to stress. The Mtb genome encodes an unusually high number of type II toxin–antitoxin (TA) systems that are thought to act as stress sensors. Type II TA systems are operons comprising adjacent genes encoding two small (∼10 kDa) proteins, a toxin and its cognate antitoxin that inhibits toxin activity through formation of a stable TA protein–protein complex. Stresses or bacteriophage infection are thought to control the level of free toxin in bacteria that harbor them (4, 5); free toxin then acts on its target inside bacterial cells. Among the 67 reported Mtb type II TA systems, 10 belong to the MazEF family (MazE-antitoxin, MazF-toxin) (6). Our group named these TA systems MazEF-mt1 through MazF-mt10 to distinguish them from *Escherichia coli* MazF. However, concurrent studies by other groups used a different naming system, simply MazEF1 through MazEF10. Therefore, the numbered names and their respective Rv numbers do not always match between nomenclatures (summarized in Table 1).

**Table 1:** Summary of *Mycobacterium tuberculosis* MazEF toxin-antitoxin systems

Woychik/Inouye name	Tuberculist/other name	Antitoxin MazE locus	Toxin MazF locus	Publications
MazEF-mt1	MazEF9	Rv2801A	Rv2801c	Sala *et al.*, 2014; Tiwari *et al.*, 2015; Zhu *et al.*, 2006; and Zhu *et al.*, 2010 (6, 8, 14, 39)
MazEF-mt2	MazEF1	Rv0456B	Rv0456A	Sala *et al.*, 2014 (6)
MazEF-mt3	MazEF6	Rv1991A	Rv1991c	Chattopadhyay *et al.*, 2022; Sala *et al.*, 2014; Schifano *et al.*, 2014 (6, 9, 59)
				Tiwari *et al.*, 2015; Zhao and Zhang, 2008; Zhu *et al.*, 2008; Zhu *et al.*, 2010 (11, 14, 39, 60)
MazEF-mt4	MazEF2	Rv0660c	Rv0659c	Sala *et al.*, 2014 (6)
MazEF-mt5	MazEF5	Rv1943c	Rv1942c	Sala *et al.*, 2014 (6)
MazEF-mt6	MazEF3	Rv1103c	Rv1102c	Hoffer *et al.*, 2017; Sala *et al.*, 2014; Schifano and Woychik, 2014; and Schifano *et al.*, 2013 (6, 10, 61, 62)
				Tiwari *et al.*, 2015; Zhu *et al.*, 2006 (8, 39)
MazEF-mt7	MazEF4	Rv1494	Rv1495	Huang and He, 2010; Sala *et al.*, 2014; and Zhu *et al.*, 2008 (6, 11, 63)
MazEF-mt8	MazEF8	Rv2274A	Rv2274c	Sala *et al.*, 2014 (6)
MazEF-mt9	MazEF7	Rv2063	Rv2063A	Barth *et al.*, 2019; Sala *et al.*, 2014; Schifano and Woychik, 2017; and Schifano *et al.*, 2016 (6, 12, 16, 64)
MazEF-mt10	MazEF10	Rv0298	Rv0299	Sala *et al.*, 2014; Kim *et al.*, 2016 (6, 65)
MazEF-mt11	mt-PemIK	3467307–3467612^a^	Rv3098A	*This work*, Chi *et al.*, 2018 (15)

a Genome position in *M. tuberculosis* H37Rv.

All bacterial MazF toxins are single-strand, sequence-specific endoribonucleases (7). The enzymatic activity for a subset of Mtb MazF toxins has been characterized *in vitro* and/or when expressed in *E. coli*: MazF-mt1 cleaves RNA at U↓AC (8), MazF-mt3 at U↓CCUU (8, 9), MazF-mt6 at UU↓CCU (10), MazF-mt7 at U↓CGCU (11), and MazF-mt9 at UU↓U (12). MazF-mt9 has been studied in the most detail; it exhibits extraordinary specificity for a single tRNA, tRNA^LysUUU^, when expressed in either *Mycobacterium smegmatis* or Mtb (13). Selective depletion of tRNA^LysUUU^ causes transcriptome-wide ribosome stalling at cognate AAA lysine codons, leading to codon-specific remodeling of the mycobacterial translatome (13).

Here, we provide evidence that the Mtb genome carries an 11th MazEF TA system, MazEF-mt11. We then precisely determine the RNA cleavage consensus sequence of the MazF-mt11 toxin and identify 16S rRNA as the sole RNA target in mycobacteria. The MazF-mt11 C↓ACCU cleavage site exclusively maps near the 3′ end 16S rRNA in mycobacteria, resulting in the removal of the terminal 11 nts carrying the anti-Shine–Dalgarno (aSD) sequence followed by translation inhibition.

## Results

### MazF-mt11 (Rv3098A) exhibits sequence and structural similarity to other MazF family members

**Figure 1:**
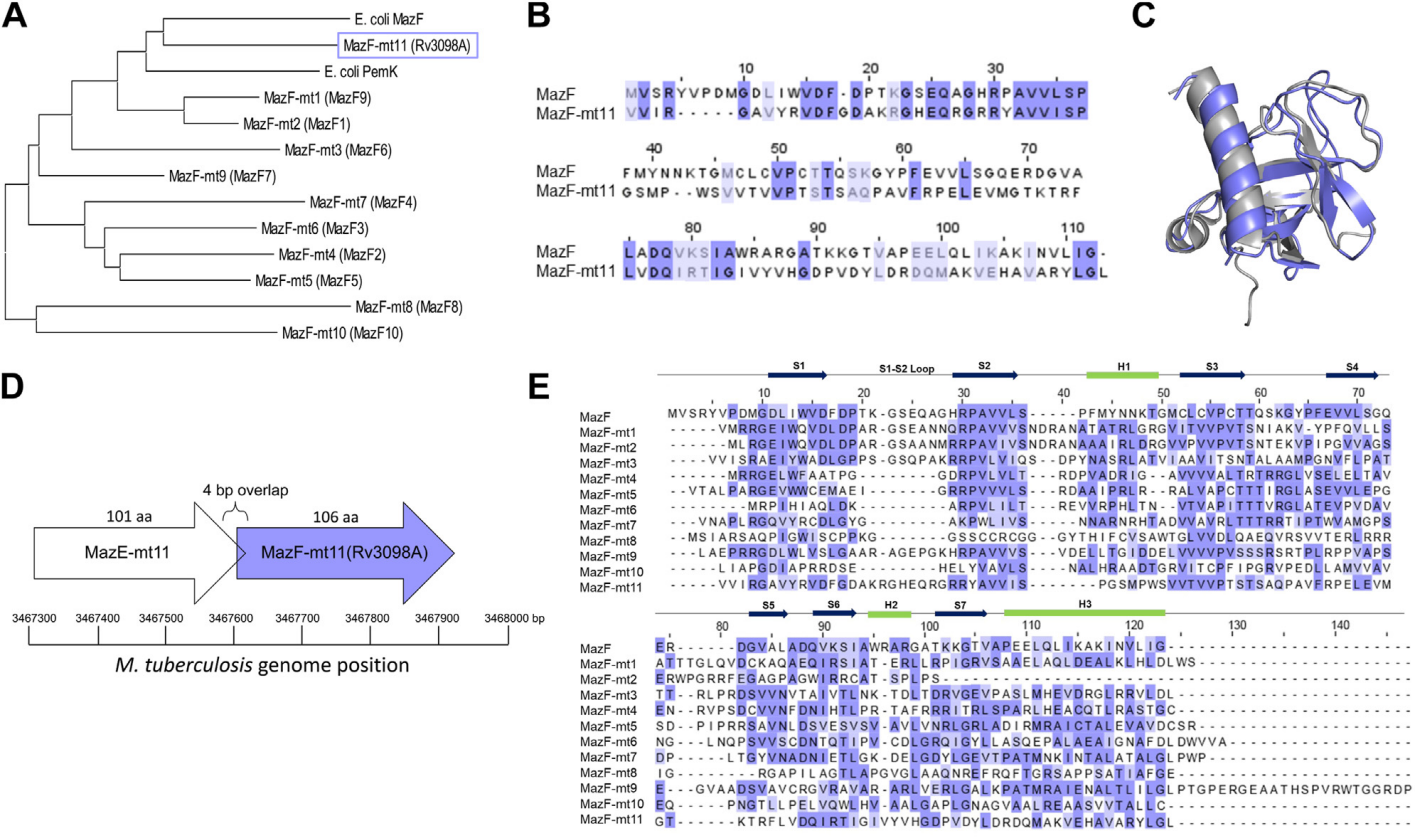
**Relationship of MazF-mt11 to the other 10 Mtb MazF toxins.***A*, phylogenetic tree with *Escherichia coli* MazF and PemK plus the 11 Mtb MazFs using the minimum evolution method with the MEGA 11 program (55). *B*, alignment of MazF-mt11 to *E. coli* MazF. *C*, high-resolution X-ray structure (Protein Data Bank code: 1UB4) of *E. coli* MazF (56) aligned with MazF-mt11 listed as “Putative toxin Rv3098A/RVBD_3098A” from the AlphaFold Protein Structure Database https://alphafold.ebi.ac.uk/. *D*, genome positions of genes encoding MazEF-mt11 proteins (amino acid length shown) in Mtb highlighting the 4 bp out of frame overlap. *E*, alignment of MazF-mt11 to the 10 published Mtb MazF paralogs using Clustal Omega (57). *Purple*, identical; *light purple*, similar (BLOSUM62 ≥1) amino acids. Secondary structures mapped above the amino acid sequences assigned from the *E. coli* MazF structure (56). Mtb, *Mycobacterium tuberculosis.*

We have been studying the MazF family of Mtb endoribonuclease toxins (8, 11, 14), of which 10 had been previously identified (6). We identified an 11th MazF-like toxin, Mtb Rv3098A (MazF-mt11), whose protein sequence is most closely related to *E. coli* MazF and *E. coli* PemK in a phylogenetic tree (Fig. 1*A*). In fact, a 2018 report characterizing some of the properties of Rv3098A referred to it as a member of the PemK family (15). The amino acid sequence identity between Rv3098A and either *E. coli* MazF (Fig. 1*B*) or PemK/Kid is equivalent (23%); in addition to identity, Rv3098A is also 18% similar to PemK and 17% to *E. coli* MazF (BLOSUM62 ≥1). These two *E. coli* toxins are so functionally similar that names of orthologs vary between MazF and PemK-like in databases such as UniProt or PANTHER. Superimposition of the *E. coli* MazF X-ray structure and AlphaFold model of Rv3098A shows a high degree of structural similarity (Fig. 1*C*), but this is also true with the PemK X-ray crystal structure. However, the predicted MazE-mt11 antitoxin sequence upstream of Rv3098A (Fig. 1*D*) is substantially more related to the *E. coli* MazE antitoxin (29% identity plus similarity) compared with the *E. coli* PemI antitoxin (5%). This coupled with the substantial shared sequence similarity between all 11 Mtb MazF toxins (Fig. 1*E*) led us to designate Rv3098A and its upstream antitoxin as Mtb MazEF-mt11 (Table 1).

### Expression of MazF-mt11 leads to growth arrest

**Figure 2:**
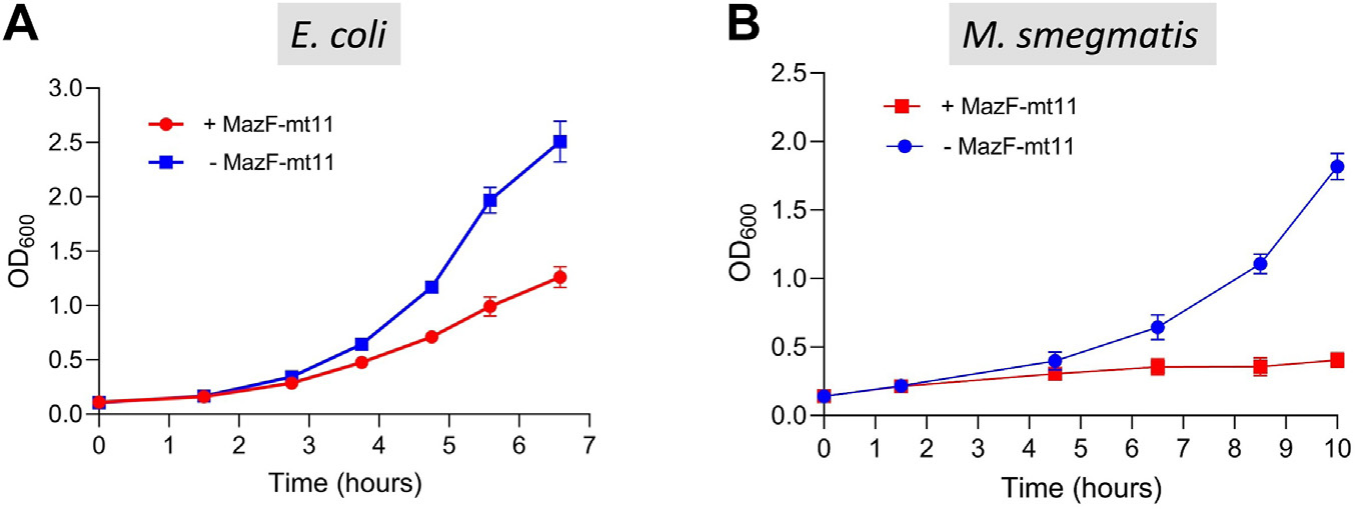
**Expression of MazF-mt11 leads to cell growth arrest.***A*, *Escherichia coli* cultures harboring pBAD33*-mazF-mt11* were grown overnight in M9 + glucose, diluted to an absorbance of 0.1 at 600 nm with M9 + glycerol (-MazF-mt11) or M9 + glycerol + 0.2% arabinose (+MazF-mt11). Error bars represent standard deviation from the average of five biological replicates. *B*, *Mycobacterium smegmatis* cultures containing the pMC1s*-mazF-mt11* plasmid were grown overnight to an absorbance of 0.1 at 600 nm, split in half and grown with added ATc (+MazF-mt11) or without ATc inducer (-MazF-mt11). Error bars represent standard deviation from the average of three biological replicates. ATc, anhydrotetracycline.

We first expressed MazF-mt11 in *E. coli* from the arabinose-inducible pBAD33 plasmid. Growth began to slow relative to the empty vector control 3 h after toxin induction, and this growth difference widened with time (Fig. 2*A*). We also expressed MazF-mt11 in *M. smegmatis—*which only has a single MazF toxin that targets tRNA^Lys^ (16)—from the anhydrotetracycline (ATc)-inducible pMC1s integrating plasmid specifically engineered for low-level expression in mycobacteria (17). Cell growth was arrested in toxin-expressing cells after 1.5 h and was sustained through 10 h (Fig. 2*B*). Therefore, as with the other Mtb MazF toxins studied to date, MazF-mt11 expression inhibits cell growth, especially in mycobacteria where the cell density essentially flatlined shortly after toxin induction.

### The MazF-mt11 cleavage consensus sequence is under-represented in *E. coli* and present only once in bacteriophage MS2

To help understand why MazF-mt11 expression leads to growth arrest and inform its mechanism of action, we sought to identify its RNA recognition sequence and cleavage position within it. We first performed northern analysis on RNA harvested from *E. coli* hybridized with oligonucleotides corresponding to three naturally abundant transcripts—*ompA*, *rpsA*, and *tufA*—encoding OmpA outer membrane protein, ribosomal protein bS1, and elongation factor Tu, respectively. If toxin expression led to reduction in any of these transcripts relative to the control, the site of cleavage could subsequently be identified by primer extension. Primer extension enables mapping of the 5′ end of an RNA after a complementary radiolabeled DNA primer is annealed to the RNA template, and this complementary DNA (cDNA) product is extended by reverse transcriptase (RT) until it reaches the end of the RNA template. The position of the RNA 5′ end can then be determined after resolving the cDNA product next to a Sanger sequencing reaction using the same primer as the RT reaction. We had previously used this approach to identify the target sequences for Mtb toxins, MazF-mt6 and VapC4 (10, 18). This strategy was especially useful for pinpointing target sequences and cleavage sites for *E. coli* endoribonuclease toxins (19, 20, 21, 22). We were surprised that we did not detect a reduction in *ompA*, *rpsA*, or *tufA* transcripts with time of expression relative to the control (data not shown), suggesting that the MazF-mt11 target sequence is not commonly found in *E. coli*.

**Figure 3:**
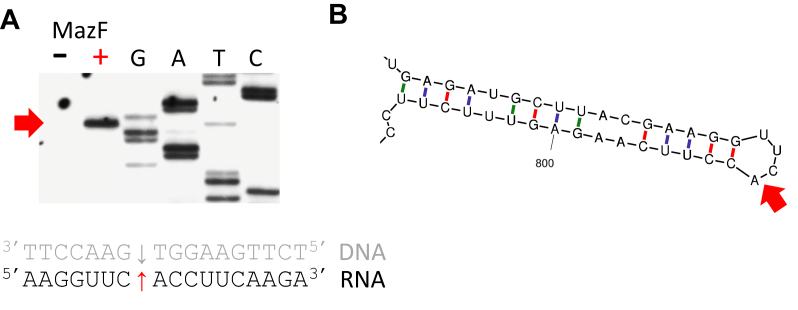
**MazF-mt11 cleaves MS2 RNA at a single site.***A*, primer extension of MS2 RNA after incubation with (+MazF) or without (-MazF) recombinant MazF-mt11 for 60 min at 37 °C showing toxin-specific band (*red arrow*, *left*). Position of cleavage within MS2 RNA (*red arrow*) is derived from the adjacent Sanger DNA sequencing ladder (read from the *bottom* up to give the 5′ to 3′ DNA sequence shown in *gray*). The toxin band aligns with the T lane band indicating that the cleavage occurs following this base (*gray arrow*). The complementary RNA sequence and cut site (*red arrow*) was deduced from the DNA sequence and matched to the MS2 sequence. *B*, MS2 RNA modeled with M-fold (58) showing position of cleavage in single-stranded loop.

As an alternative, recombinant MazF-mt11 was incubated with the MS2 enterobacteriophage genomic RNA. This 3569 nt commercially available single-stranded RNA substrate serves as a relatively long template useful for identification of *in vitro* toxin cleavage sites (11, 12, 21, 22, 23, 24, 25, 26). We performed primer extensions using oligonucleotides spanning the entire MS2 RNA but only identified a single cleavage site for MazF-mt11 (Fig. 3*A*). Detailed studies using a variety of RNA and RNA–DNA templates indicated that recombinant *E. coli* MazF cleaves single-stranded RNA at ACA sites (27). In agreement, in the MS2 secondary structure MazF-mt11 cleaves between a C↓A that maps to a loop region (Fig. 3*B*). Since there are 202 CA sequences in MS2, this toxin likely recognizes a longer, more complex RNA sequence that includes a CA and/or requires the RNA consensus sequence/cleavage site to be in the context of a specific secondary structure. The latter is true for Mtb MazF-mt9 recognition of the tRNA^LysUUU^ anticodon stem and loop; it not only cleaves the anticodon sequence at U↓UU within the single-strand anticodon loop but also requires the intact anticodon stem structure for cleavage (12, 13).

### MazF-mt11 cleaves RNA at CACCU sequences

**Figure 4:**
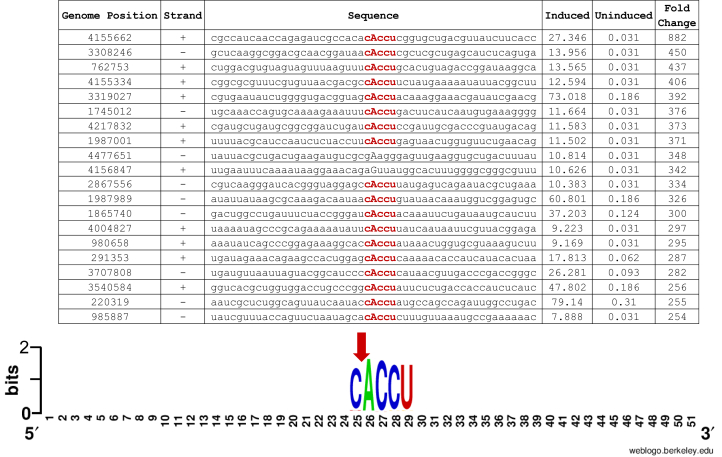
**The MazF-mt11 RNA cleavage consensus sequence is CACCU.***A*, we used MORE RNA-seq (9) to identify the 5 nt RNA recognition sequence enriched 100-fold or more in 5′-OH RNA-seq libraries prepared from RNA harvested from *Escherichia coli* cells after 3 h with or without MazF-mt11 induction. *Upper panel* shows top 20 RNA hits of 167 total with resulting consensus sequence in *red*; MazF-mt11 cleavage site occurs 5′ of the uppercase *A*, *lower panel* shows resulting WebLogo (53). MORE, Mapping by Overexpression of an RNase in *E. coli*.

We used a specialized RNA-seq method we developed, MORE (Mapping by Overexpression of an RNase in *E. coli*) RNA-seq, as a powerful genome-scale tool to identify the RNA sequence needed for MazF-mt11 recognition as well as determine the cleavage position to single nt resolution (9). MazF-mt11 was expressed from the arabinose-inducible pBAD33 plasmid in an *E. coli* genetic background lacking six endoribonuclease TA systems, including MazEF. RNA was then harvested from the toxin-expressing and empty vector control cells 3 h postinduction and used for MORE RNA-seq (Table S1). The upper portion of Figure 4 depicts the top 20 *E. coli* RNAs exhibiting the highest fold increase in cleavage by MazF-mt11 relative to the control, revealing a clear C↓ACCU consensus in bold red text. The lower portion of Figure 4 shows the corresponding WebLogo derived from all 167 RNAs that met our ≥100 fold-change cutoff. Therefore, we conclude that the RNA target for MazF-mt11 is a CACCU sequence, and cleavage occurs between the C↓A. This finding is consistent with our identification of only one MS2 cleavage site; the CACCU consensus sequence occurs just once in this template, exactly where we mapped the cut site (Fig. 3). Identification of CACCU as the consensus sequence of MazF-mt11 also explains the stability of *E. coli ompA*, *rpsA*, and *tufA* after toxin expression; none of these transcripts contains a CACCU sequence.

### MazF-mt11 cleaves a single CACCU-containing RNA target in mycobacteria preceding the aSD sequence at the 3′ end of 16S rRNA

We have previously demonstrated that MORE RNA-seq is an excellent genome-scale method for determining the cleavage consensus sequence and position of cleavage for a mycobacterial endoribonuclease toxin (9, 12). However, the identification of the actual mycobacterial RNA target requires expression in a mycobacterial host (13, 28). For example, MORE RNA-seq correctly pinpointed the toxin consensus and cleavage site as UU↓U for MazF-mt9 with cleavage of six different *E. coli* tRNAs, including tRNA^LysUUU^ (12). We subsequently discovered that the toxin must be expressed in the appropriate host to identify the true target in Mtb—only tRNA^LysUUU^—indicating that the recognition was isoacceptor specific (the other lysine tRNA, tRNA^LysCUU^, was not cleaved) (13). Therefore, while the consensus sequence and cleavage site derived from MORE RNA-seq has always been reliable, identification of the true RNA target requires expression in the appropriate host.

**Figure 5:**
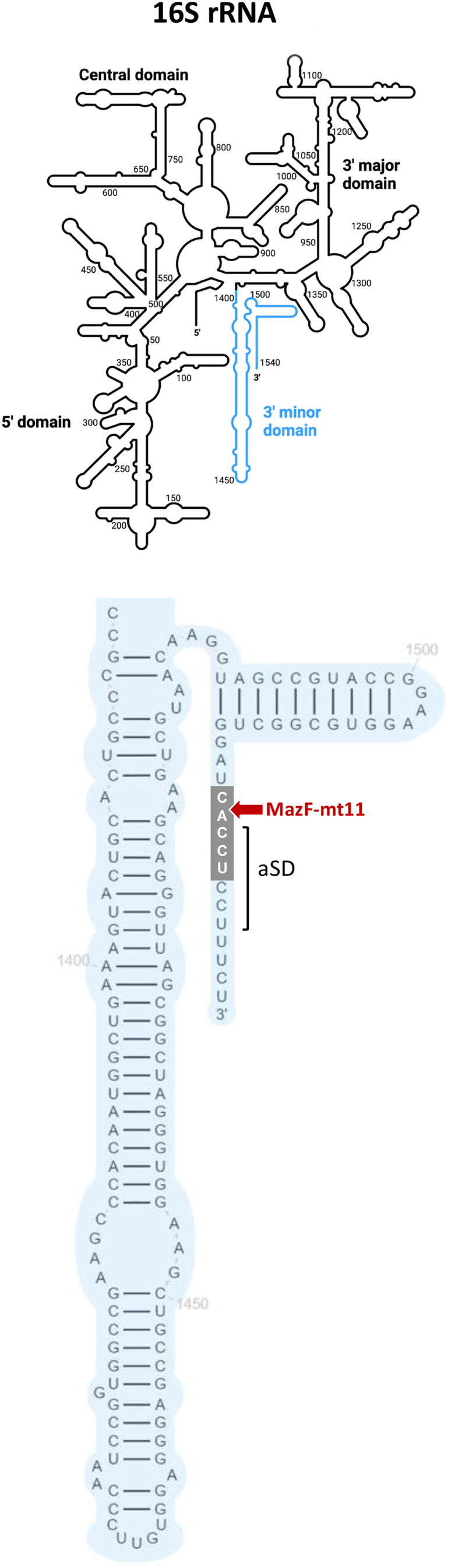
**The MazF-mt11 cleaves mycobacterial 16S rRNA 11 nt from its 3′ end.***Top*: general schematic of the entire bacterial 16S rRNA from BioRender (3′ minor domain highlighted in *blue*). *Bottom*: detailed sequence and secondary structure of the mycobacterial 16S rRNA 3′ minor domain comprising helices 44 and 45 and the single-stranded 3′ end highlighting the MazF-mt11 cleavage site (*gray box*) and the CCUCCU aSD sequence (*bracketed*). The *Mycobacterium smegmatis* and *Mycobacterium tuberculosis* sequences from helix 45 to the 3′ end are identical.

We expressed MazF-mt11 in *M. smegmatis* using the same ATc-inducible pMC1s expression vector as in Figure 2*B* and performed 5′-OH RNA-seq from RNA harvested from toxin-induced and -uninduced cells (Table S2). In contrast to MORE RNA-seq (Fig. 4) only a single target near the 3′ end of the 16S rRNA (Fig. 5) had the CACCU consensus sequence; the position of the cleavage site within this 5 nt sequence also matched that in *E. coli*, C↓ACCU. Since the sequence helix 45 to the 3′ end of 16S rRNA are identical in *M. smegmatis* and Mtb (Fig. 5), this experiment enabled us to pinpoint the sole Mtb MazF-mt11 target. Notably, C↓ACCU cleavage results in removal of the CCUCCU aSD sequence that can base-pair with the Shine–Dalgarno (SD) sequence in mRNAs to help align the ribosome with the start codon to enable efficient translation initiation (Fig. 5).

We also identified an unusual increase in cleavage within intragenic and intergenic IS*1549* insertion sequences (29) coincident with MazF-mt11 expression. The toxin is not directly responsible for the IS*1549* cleavage because there is no C↓ACCU consensus at those cut sites, nor is there any clear consensus sequence favored (Fig. S1). The National Center for Biotechnology Information (NCBI) annotates these internal IS*1549* cleavages as “ND” when inserted within annotated genes or as “IS*1549*” when inserted outside the genes in the “Description” column of Table S2. We posit that these indirect, downstream events are the consequence of an unknown RNase that generates a 5′-OH that is activated by the ribosome stress imparted by MazF-mt11. Although *M. smegmatis* RNase E has an essential role in mRNA degradation, it is not a candidate because it generates a 5′-monophosphate upon cleavage and exhibits a preference for cleavage before a cystine (30). It is known that bacterial stress generally leads to a “transposition burst” (31, 32). However, it is unclear if these enhanced 5′-OH-generating cleavage events reflect an increase in IS*1549* transposition followed by cleavage and recycling or simply an increase in cleavage and recycling of existing IS*1549* sequences.

### MazF-mt11 cleavage of the terminal 11 nts of 16S rRNA leads to translation inhibition

**Figure 6:**
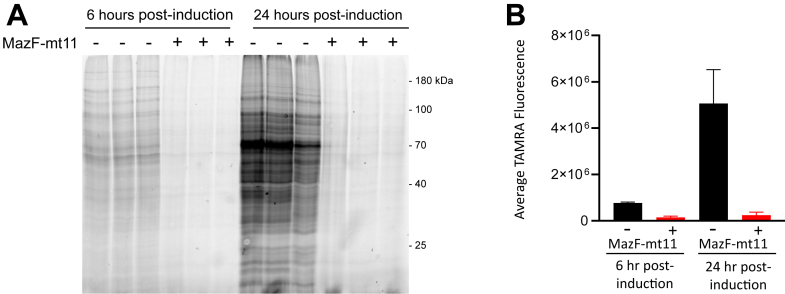
**MazF-mt11 removal of the aSD sequence from 16S rRNA leads to translation inhibition.***A*, MazF-mt11 was induced for 6 or 24 h in *M. smegmatis* cells followed by labeling with the methionine mimetic AHA for 3 h and visualized upon alkyne-TAMRA conjugation. Each lane was normalized to 10 μg of total protein. *B*, Whole lane TAMRA fluorescence signals were quantified from triplicates shown, then averaged and plotted. aSD, anti-Shine–Dalgarno sequence; TAMRA, tetramethylrhodamine; AHA, azidohomoalanine.

To determine if removal of the aSD sequence influences translation, we used a click chemistry approach for metabolic labeling to monitor *de novo* protein synthesis in *M. smegmatis* cells with or without MazF-mt11 expression. Incorporation of the azide-containing Met mimetic azidohomoalanine (AHA) enabled fluorescent visualization of AHA-containing proteins upon coupling to the alkyne tetramethylrhodamine. Loss of the aSD sequence severely impaired new protein synthesis 6 and 24 h after toxin induction (Fig. 6*A*), with new protein synthesis levels dropping to 19% relative to the uninduced control after 6 h and 5% after 24 h (Fig. 6*B*).

## Discussion

Mtb MazF toxins have been specifically linked to the nonreplicating persistent state characteristic of latent TB and the general Mtb stress response. First, MazF activity in other bacteria is known to play a role in the stress response and antibiotic tolerance (33, 34); MazF transcripts are also upregulated in drug-tolerant Mtb cells (35). Second, expression of a single Mtb MazF toxin can lead to a growth-arrested state in Mtb, *M. smegmatis*, or *E. coli* cells (8, 10, 12). Third, MazF transcripts are upregulated upon nutrient starvation (36, 37, 38). Finally, some MazFs are required for virulence in guinea pigs (39).

Here, we characterized the RNA recognition site, the precise position of cleavage, and the lone RNA target for an 11th MazF toxin in Mtb toward the goal of understanding how these abundant type II endoribonuclease toxins facilitate stress protection. We utilized tools developed in our laboratory (9) to first reveal the C↓ACCU site when MazF-mt11 was expressed in *E. coli*. We next pinpointed the target of MazF-mt11 to a single site within 16S rRNA by expressing this toxin in *M. smegmatis* (which does not have an MazF-mt11 counterpart and whose 16S rRNA helix 45 and 3′ end sequences are identical to Mtb). In subsequent studies following our original 2014 paper on the utility of MORE RNA-seq using an *E. coli* host (9) we learned that identification of the true RNA target requires toxin expression in its natural host (12, 23, 28, 40). MazF-mt11 MORE RNA-seq revealed a near-perfect C↓ACCU WebLogo within the 167 RNA targets whose cleavage was enriched ≥100-fold relative to the uninduced control (Table S1). However, there is limited physiological insight provided by mRNAs and other RNAs comprising MazF-mt11 targets in *E. coli*. Nevertheless, to date, the utility of MORE RNA-seq for its ability to accurately and reliably reveal the toxin cleavage site and consensus sequence is sound. As mentioned previously, MORE RNA-seq following expression of MazF-mt9 in *E. coli* generated a strong UU↓U WebLogo from cleavage of 48 RNAs enriched ≥50-fold relative to the uninduced control (12). Yet, when MazF-mt9 is expressed in Mtb, it only recognizes a single RNA target, tRNA^LysUUU^, which is cleaved at the UU↓U comprising the anticodon (13). This exquisite toxin specificity is also recapitulated here for MazF-mt11. Following 5′ RNA-seq in *M. smegmatis*, the only RNA cleaved at the C↓ACCU consensus sequence was 16S rRNA in a region that partially overlapped with the aSD sequence. In fact, both copies of 16S rRNA (rrsA, rrsB) in *M. smegmatis* were identified in our dataset (Table S2). Although the Mtb and *M. smegmatis* helix 45 and 3′ end including the aSD sequence are identical, and their 16S rRNA sequences are over 95% identical overall, we cannot exclude the possibility that there are additional targets in Mtb.

The position of 16S rRNA cleavage by MazF-mt11 is intriguing. The canonical function of the conserved aSD CCUCCU sequence of the 30S subunit is to bind to the complementary GA-rich SD sequence present upstream in most mRNAs, direct the ribosome to the start codon, and promote translation initiation. By selectively targeting removal of the critical aSD sequence at the 3′ end of 16S rRNA in the ribosome, the strong MazF-mt11 growth arrest phenotype in mycobacteria might be due to inefficient translation initiation. Failure to translate and meet thresholds of just one essential protein would inhibit growth, and likely many essential protein demands are not met when ribosomes cannot efficiently pair with cognate SD sequences in leadered mRNAs. Curiously, although many antibiotics perturb the ribosome, none are known to target the 16S rRNA helix 45 and terminal nts at the 3′ end that include the aSD sequence.

Chi *et al.* (15) not only studied the basic properties of MazEF-mt11 (referring to it instead as an Mtb PemIK TA system) but also expressed the toxin in mycobacteria followed by RNA-seq to survey the transcriptome following toxin expression. Although they did not know the RNA target of MazF-mt11, only 5.6% of transcripts were downregulated (371 of 6625 mRNAs), which fell into a broad range of functional categories; several of these downregulated genes were essential (15). However, proteomic studies would be more informative given the role of the aSD in protein synthesis. To this end, we demonstrated that new protein synthesis is substantially reduced after 6 h of toxin expression and almost completely blocked 24 h postinduction (Fig. 6, *A* and *B*), consistent with the strong growth arrest phenotype following toxin induction in *M. smegmatis* (Fig. 2*B*). The marginal reduction in global transcript stability reported by Chi *et al.* might indicate that toxin-mediated effects on protein synthesis are not strongly coupled with transcription or result from the accumulation of suppressor mutations in the host cell and/or the toxin coding sequence because the authors induced toxin expression very late (near stationary phase).

There is a dearth of information on the significance of the aSD in bacterial translation (41); most studies address how the SD influences translation initiation (reviewed in Ref. (42)). Curiously, leaderless transcripts that begin with the translation start codon and lack an SD are unusually abundant (∼25% of mRNAs) in Mtb and *M. smegmatis* (38, 43, 44) but rare in *E. coli* (45). A survey of 12,495 16S rRNA 3′ ends revealed that the CCUCCU aSD sequence is variable (closely or distantly related) or completely absent in 128 bacteria (46), indicating that there are aSD-independent mechanisms of translation initiation.

Cortes *et al.* (38) also studied the fate of Mtb transcripts subjected to starvation; 75% of TA systems—including seven Mtb MazFs—were upregulated, but the Rv number for MazF-mt11 was not in this dataset. An earlier study linking *E. coli* MazF removal of the aSD sequence to selective leaderless mRNA transcription (45, 47) has not been supported by rigorous follow-up studies (48). Although ∼14% of transcripts for annotated genes are leaderless in *M. smegmatis* (49) and Mtb (38, 44), it is unclear from our metabolic labeling experiment if new protein synthesis that significantly diminishes with time (19% and 5% relative to the control after 6 and 24 h of toxin expression, respectively, Fig. 6, *A* and *B*) represents translation from ribosomes lacking an aSD or simply from normal ribosomes whose 16S rRNA was not cleaved by MazF-mt11. Since cells expressing MazF-mt11 are growth arrested (Fig. 2*B*), this toxin may participate in the establishment and/or maintenance of the nonreplicating persistent state characteristic of latent TB infection. The physiological triggers of MazF-mt11 activation in Mtb and its role in TB infection are not yet known but represent an area of active study in our laboratory.

## Experimental procedures

### Strains, plasmids, and reagents

The *E. coli* strain BW25113Δ6 (*lacI*^q^ *rrnB*_T14_ Δ*lac-Z*_WJ16_ *hsdR514* Δ*araBAD*_AH33_ Δ*rhaBAD*_LD78_ Δ*chpBIK* Δ*dinJ-yafQ* Δ*hipBA* Δ*mazEF* Δ*relBE* Δ*yefM-yoeB*) (21) (obtained from the Masayori Inouye laboratory; validated upon performing 5′ RNA-seq in this study) was used for all *E. coli* growth profile and RNA cleavage studies. The *E. coli* strain Mach1-T1 (F^−^Δ*rec*A1398 *end*A1 *ton*A ϕ80(*lacZ*)ΔM15 Δ*lac*X74 *hsd*R(r_κ_^–^, m_κ_^+^); Invitrogen) was used for all cloning experiments. The *E. coli* strain BL21(DE3) (F^–^*omp*T *hsdS*_β_(r_β_-m_β_) *dcm gal* (DE3) *ton*A; Novagen) was used for recombinant protein expression. The plasmids pBAD33 (50) and pET-28a were used for expression in *E. coli*. All *E. coli* liquid cultures were grown at 37 °C in M9 minimal medium supplemented with either 0.2% glucose or 0.1% glycerol. Toxin was expressed *via* an arabinose-inducible promoter in pBAD33-*mazF*-*mt11*. The working concentrations of chloramphenicol and kanamycin were 25 μg/ml and 50 μg/ml, respectively.

The *M. smegmatis* strain mc^2^155 (obtained from the Robert Husson laboratory, Boston Children’s Hospital; validated upon performing 5′ RNA-seq in this study) was used for all *M. smegmatis* growth profiles. For 5′ RNA-seq studies, we used mc^2^155 with an RNase J deletion (*rnj*, MSMEG_2685) (13) as well as wildtype mc^2^155 (data not shown). The MazF-mt11 5′ RNA-seq datasets were essentially identical in both strain backgrounds. The plasmid pMC1s was used for expression in *M. smegmatis* (17). All *M. smegmatis* liquid cultures were grown at 37 °C in 7H9 Middlebrook media supplemented with 0.05% Tween-80, 0.5% bovine albumin, 0.2% glucose, and 0.085% NaCl. Toxin was expressed *via* a tetracycline-inducible promoter in pMC1s-*mazF-mt11*. The working concentration of kanamycin was 25 μg/ml.

### Preparation of recombinant His_6_-MazF-mt11

pET-28a-*mazF-mt11* BL21(DE3) transformants were used to inoculate 1 l of M9 liquid medium and grown to an absorbance of 0.6 at 600 nm. Transformants were induced with a final concentration of 1 mM isopropyl-β-d-thiogalactopyranoside, and protein was expressed for 2.5 h. Cells were disrupted by sonication, and extracts were purified by nickel–nitrilotriacetic acid affinity chromatography (Qiagen).

### *In vitro* primer extension analysis of MS2 enterobacteriophage genomic RNA

MS2 enterobacteriophage genomic RNA was incubated with purified His_6_-MazF-mt11, and primer extension analysis was performed as previously described (9). The oligonucleotide NWO1726, 5′-GCTGGATACGACAGACGGCCATC-3′ was used for both primer extension and sequencing reactions.

DNA oligonucleotides were radiolabeled at the 5′ end by treating with T4 polynucleotide kinase (New England Biolabs) and [γ^32^P]ATP (Revvity) for 90 min at 37 °C. Cleavage reactions using 0.8 μg (0.77 pmol) of MS2 RNA (Roche/MilliporeSigma) as a substrate were incubated with or without 10 μM of MazF-mt11 for 60 min at 37 °C. Primer extension reactions were then performed at 50 °C for 60 min with 1 pmol of 5′-end-radiolabeled primer, 10 U of RNase inhibitor (Roche Applied Science), and 2.5 U AMV RT (New England Biolabs) in the supplied enzyme buffer. For DNA sequencing reactions, 1 pmol of 5′-end radiolabeled primer was annealed to 0.8 μg (0.77 pmol) of MS2 RNA for 30 s at 80 °C in the supplied AMV RT buffer, then allowed to cool to room temperature. The sequencing reactions were then performed at 50 °C for 1 h using 10 U of RNase inhibitor (Roche) and 1 U of AMV RT (New England Biolabs) in place of Sequenase DNA polymerase, as well as dNTPs and ddNTPs from the Sequenase version 2.0 DNA sequencing kit (ThermoFisher) at the concentration recommended by the manufacturer. Reactions were electrophoresed on a 6% (w/v) polyacrylamide 7 M urea gel and visualized by autoradiography.

### RNA isolation for 5′-OH RNA-seq

Total RNA was isolated from *E. coli* freshly transformed with either pBAD33 or pBAD33-*mazF*-*mt11* and *M. smegmatis* with pMC1s-*mazF-mt11* grown to midlogarithmic phase. *E. coli* cultures were grown to an absorbance of 0.3 at 600 nm, after which arabinose was added to a final concentration of 0.2% and the cultures were grown for an additional 3 h. Cells were harvested when the induced cultures started to show a growth separation from the cells containing empty pBAD33. *M. smegmatis* cultures were grown to an absorbance of 0.1 at 600 nm, after which the culture was split into equal portions and ATc was added to one portion to a final concentration of 200 ng/ml and the cultures were grown for an additional 5 h. Cells were pelleted at 3000*g* at 4 °C for 10 min, resuspended in TRIzol Reagent (Invitrogen), and lysed in a Precellys Evolution with 0.1 mm silica beads at 10,000 rpm three times for 30 s each time. Lysates were precipitated with ethanol and added to a Zymo-Spin IIC column. After an initial wash with Direct-zol RNA Wash Buffer, genomic DNA was digested with 5 U of DNase I. The RNA was purified following the Direct-zol RNA Miniprep protocol (Zymo Research). To further purify the RNA, RNA was eluted from the column with nuclease-free water, treated with 2 U of TURBO DNase (Invitrogen) for 30 min at 37 °C, and purified using ethanol, a Zymo-Spin IIC column, and the Direct-zol RNA PreWash and Wash buffers as directed by the Direct-zol RNA Miniprep protocol. RNA was eluted from the column with nuclease-free water, and the quantity and quality of the RNA checked using an Eppendorf BioSpectrometer.

### Preparation of RNA for high-throughput sequencing

RNA was prepared for sequencing as previously described (Schifano *et al.*, 2016 (12)), with some slight modifications. Since rRNA is a potential target of MazF toxins, we did not deplete rRNA before cDNA preparation. RNA (3 μg) was treated with 1 U of Terminator 5′-Phosphate-Dependent Exonuclease (LGC Biosearch Technologies). This process removed RNAs with a 5′-monophosphate (5′-P) but left RNAs with a 5′-hydroxyl (5′-OH). Then, the RNAs with a 5′-OH were phosphorylated with 50 U of T4 PNK to create 5′-P ends that are suitable for ligation to a 5′ adapter. Next, these RNAs were ligated to the Illumina 5′ RNA adapter (5′-GUUCAGAGUUCUACAGUCCGACGAUCNNNNNN-3′). Ligation reactions contained 5′-P RNA, 30 pmol Illumina 5′ adapter, and 10 U T4 RNA Ligase I (New England Biolabs) and were incubated at 16 °C for 20 h. Excess 5′ adapter was separated from the 5′ adapter-ligated RNAs by running the ligation reactions on a 1 mm 6% Tris–borate–EDTA (TBE)–urea gel and gel-excising RNAs that migrated above the free 5′ adapter. Then, cDNA libraries were generated *via* reverse transcription using a primer with nine degenerate nucleotides at the 3′ end, Illumina (5′-GCCTTGGCACCCGAGAATTCCANNNNNNNNN-3′). To perform the annealing step, 500 to 600 ng of 5′ adapter-ligated RNA was mixed with 500 ng of RT primer and incubated at 85 °C for 3 min and then at 4 °C for 1 min. Then reverse transcription was performed with 200 U SuperScript IV Reverse Transcriptase (Invitrogen/ThermoFisher), dNTPs, and the RNA with the annealed primer. The mixture was incubated at 25°C for 5 min and then 55 °C for 30 min. After the reverse transcription, the RT enzyme was inactivated at 80 °C for 10 min. Then, 10 U of RNase H (Ambion/ThermoFisher) was added, and the mixture was incubated at 37 °C for 30 min to digest the RNA from the DNA–RNA duplexes. The samples were electrophoresed on a 1 mm 10% TBE–urea gel, and cDNAs from 80 to 500 nts were excised from the gel. The cDNA libraries were PCR amplified using reagents from a TruSeq Small RNA Sample Prep Kit (Illumina). The PCR had an initial denaturation step of 30 s at 98 °C, 12 cycles of annealing as follows: denaturation—10 s at 98 °C; annealing—20 s at 62 °C; extension—10 s at 72 °C, and a final extension for 7 min at 72 °C. The amplified DNA was run on a 1.0 mm 10% TBE-PAGE gel, and DNA from 120 bp to 450 bp was excised from the gel and sequenced with the Illumina platform.

### 5′ RNA-seq data analysis

Sequencing reads for which the first 25 bases mapped with zero mismatches to the *E. coli* K12 substrain MC 4100 or *M. smegmatis* strain mc^2^155 genome (accession numbers: HG738867 and CP000480, respectively) in the NCBI GenBank genome browser (51) were identified using Bowtie (version 1.3.1) (52). We determined the fold-change difference between MazF-induced and empty vector control for *E. coli* or uninduced for *M. smegmatis* by dividing induced sequencing read counts by control read counts and considered all hits with a ≥5-fold increase. We further sifted the data to only include reads that represented local maxima within a 50-base window spanning 25 bases upstream and downstream. If redundant sequences caused cleavage sites to map to more than one position in the genome, then we only counted the sequence surrounding each site of cleavage once. Finally, we aligned the significant reads from each MazF using WebLogo (53) to determine the consensus sequence. All 5′ RNA-seq hits with a fold change of ≥100 were considered for this *E. coli* WebLogo; no restrictions were set for *M. smegmatis* data as there were only two identical rRNA (rrsA/rrsB) hits with the C↓ACCU consensus sequence in the dataset.

## Labeling of newly synthesized proteins

To assess new protein synthesis in *M. smegmatis* after induction of MazF-mt11, triplicate samples were induced from absorbance of 0.1 at 600 nm, and AHA (Sigma) was added after 6 and 24 h, to a final concentration of 50 μM and cultures were incubated for another 3 h. To extract AHA-labeled proteins, cells were pelleted, resuspended in lysis buffer (2% CHAPS and 8 M urea), and lysed using the Precellys Evolution homogenizer as described previously for RNA isolation. The lysate was centrifuged, and the proteins from the supernatant were linked to an alkyne-containing fluorophore (tetramethylrhodamine) using the Click-&-Go Protein Reaction Buffer Kit (Vector Laboratories). A total of 10 μg of protein from each sample were resolved on a 17% SDS-PAGE gel, imaged with a Typhoon FLA 9500 (GE Healthcare) image system, and quantified using Bio-Rad Image Lab 6.1.

## Data availability

The RNA-seq data generated in this study have been deposited in the NCBI Sequence Read Archive under accession number PRJNA1210630.

## Supporting information

This article contains supporting information (Tables S1, S2 and Fig. S1) (54).

## Conflict of interest

The authors declare that they have no conflicts of interest with the contents of this article.
