# Supplementary material for: Targeted removal of the 16S rRNA anti-Shine–Dalgarno sequence by a Mycobacterium tuberculosis MazF toxin
Source: J Biol Chem. 2025 May 30;301(7):110323. doi: 10.1016/j.jbc.2025.110323 (PMC12274833; doi:10.1016/j.jbc.2025.110323)
Supplement: Supplementary Figure 1 [file mmc1.docx]

**Supplemental Fig. 1. Cleavage sites within IS*1549* insertion sequences upon MazF-mt11 expression do not exhibit a sequence preference.** Frequency logo, A, and WebLogo, B, created using kpLogo (1) showing no sequence preferences at or near the 5’-OH cleavage site (between positions 24-25).

1. Wu, X., and Bartel, D. P. (2017) kpLogo: positional k-mer analysis reveals hidden specificity in biological sequences. *Nucleic Acids Res*. **45**, W534-W538
